# Human Leukocyte Antigens-B and -C Loci Associated with Posner-Schlossman Syndrome in a Southern Chinese Population

**DOI:** 10.1371/journal.pone.0132179

**Published:** 2015-07-10

**Authors:** Jun Zhao, Tianhui Zhu, Wenjie Chen, Bao Jian Fan, Liumei He, Baocheng Yang, Zhihui Deng

**Affiliations:** 1 Shenzhen Key Laboratory of Ophthalmology, Shenzhen Eye Hospital Affiliated to Jinan University, Shenzhen, Guangdong, People’s Republic of China; 2 School of Ophthalmology & Optometry Affiliated to Shenzhen University, Shenzhen, Guangdong, People’s Republic of China; 3 Massachusetts Eye and Ear Infirmary, Harvard Medical School, Boston, MA, United States of America; 4 Immunogenetics Laboratory, Shenzhen Blood Center, Shenzhen, Guangdong, People’s Republic of China; University of Birmingham, UNITED KINGDOM

## Abstract

The etiology of Posner-Schlossman syndrome (PSS) remains unknown. The association of human leukocyte antigens (HLA) allelic diversity with PSS has been poorly investigated. To evaluate the association of allelic polymorphisms of class I HLA-A, -B and -C and class II HLA-DRB1 and -DQB1 with PSS, 100 unrelated patients with PSS and 128 age- and ethnically matched control subjects were recruited from a southern Chinese Han population. Polymorphisms in exons 2–4 for HLA-A, -B, -C loci, exon 2 for HLA-DRB1 and exons 2,3 for HLA-DQB1 were analyzed for association with PSS at allele and haplotype levels. The allele frequency of HLA-C*1402 in PSS patients was significantly higher than that in controls (*P* = 0.002, OR = 4.12). This association survived the Bonferroni correction (*P_c_* = 0.04). The allele frequency of HLA-B*1301 in PSS patients was lower than that in the control group (*P* = 0.003, OR = 0.21), although this association did not survive the Bonferroni correction (*P_c_* = 0.16). In PSS patients, the haplotype frequencies of HLA-A*1101~C*1402 and B*5101~C*1402 were higher than that in controls (*P* = 0.03, OR = 4.44; *P* = 0.02, OR = 3.20; respectively), while the HLA-B*1301~C*0304 was lower than that in controls (*P* = 0.007, OR = 0.23), although these associations did not survive the Bonferroni correction (*P_c_* > 0.16). This study for the first time demonstrated that polymorphisms at the HLA-B and HLA-C loci were nominally associated with PSS in the southern Chinese Han population. Our results suggest that HLA-C*1402, A*1101~C*1402 and B*5101~C*1402 might be risk factors for PSS, whereas HLA-B*1301 plus B*1301~C*0304 might be protective factors against PSS, but even larger datasets are required to confirm these findings. Findings from this study provide valuable new clues for investigating the mechanisms and development of new diagnosis and treatment for PSS.

## Introduction

In 1948, Posner and Schlossman first reported glaucomatocyclitic crisis (GCC) and described the key features of this syndrome. So GCC was usually called as Posner-Schlossman syndrome (PSS) [1]. PSS is known as a self-limiting eye disease with benign prognosis, but the glaucomatous impairment of visual function in some patients with PSS has been increasingly reported [2,3].

The etiology of PSS has remained to be elucidated. Several factors including autonomic defect, allergic conditions, viral infection and DNA oxidative damage of the trabecular meshwork have been postulated to contribute to the development of PSS [4–9]. The vascular theory considers glaucomatous optic neuropathy to be a consequence of insufficient ocular blood supply, which can be a consequence of vascular endotheliopathy [10–13].

PSS has some clinical features of glaucoma and uveitis, such as the elevated intraocular pressure (IOP) and keratic precipitates (KPs). Human leukocyte antigens (HLA) class I and class II molecule were demonstrated to be significantly associated with uveitis and glaucoma [14–26]. Many kinds of uveitis including anterior uveitis, multiple sclerosis-associated intermediate uveitis, birdshot chorioretinitis and sympathetic ophthalmia have been associated with class I HLA [14–22]. Acute anterior uveitis (AAU) is the most common form of intraocular inflammation, and approximately 50% of cases were HLA-B27 positive [14]. HLA-B27-associated AAU was a distinct clinical entity that had wide-ranging medical significance due to its ocular, systemic, immunologic, and genetic features [15–17]. HLA-A29-positive can serve as one of the important indicators for clinical diagnosis of birdshot chorioretinitis (BSCR). BSCR accounts for approximately 7% of posterior uveitis. BSCR was strongly associated with HLA-A29, and 85% to 95% of patients with BSCR possessed HLA-A29, with an overall estimate of 90% in Caucasian or White French [18–22]. It is well known that some uveitis syndromes, such as Behçet disease (BD) and Vogt-Koyanagi-Harada disease [14,15], are associated with certain HLA types. Kang et al [23] reported that HLA-A*0207, A*2601 and A*3004 were associated with increased risk of BD, while HLA-A*3303 was associated with decreased risk of BD. HLA-A*0207, A*2601, and A*3004 were associated with skin lesions and arthritis, with uveitis, and with vascular lesions, genital ulcers, and a positive pathergy test, respectively. HLA-DR3 significantly increased in Mexican primary open angle glaucoma (POAG) patients compared with controls [24]. HLA-DRB1*0407~DQB1*0302 haplotype have been significantly associated with POAG in Mexicans [25]. DR11 and DQ1 alleles have been reported to be significantly associated with POAG in an Italian population [26].

The literature on the association between PSS and HLA is extremely limited. Only one study by Hirose et al [27] reported that HLA-Bw54 and haplotype HLA-Bw54-Cw1 showed significant association with PSS in a Japanese population, suggesting that immunogenetic factors may play an important role in the pathogenesis of PSS. However, the number of PSS patients in the Japanese study (n = 22) was limited and only classical class I HLA (HLA-A,-B and-C) were serologically typed at low resolution level [27].

To elucidate the role of HLA in the pathogenesis of PSS and provide potential molecular markers for clinical diagnosis of PSS, we genotyped class I and II HLA at high resolution level by sequencing based typing (SBT) in a southern Chinese sample of 100 patients with PSS and 128 control subjects and investigated the association of HLA allelic diversity with PSS.

## Materials and Methods

### Patients and control subjects

The study protocol was approved by the local institutional ethnics committee of Shenzhen Eye Hospital and was in accordance with the tenets of the Declaration of Helsinki. Written informed consent was obtained from all study participants after the nature of the study was explained. One hundred unrelated patients with PSS attending the Clinic of Shenzhen Eye Hospital were randomly enrolled between 2011and 2014. A presumptive diagnosis of PSS was made based on the following criteria [1,10,28]: (1) more commonly in the individuals aged 20–50 years with an equal gender distribution; (2) a history or record of unilateral recurrent transient episodes of elevated IOP higher than 21 mmHg and duration of attack varying from a few hours to several weeks. IOP may reach 40 mmHg or more, and spontaneous remission usually occurs; (3) mild cyclitis: a few white KPs accumulating in the lower half of the cornea, and minimal flare and/or a few inflammatory cells in the anterior chamber; (4) open iridocorneal angle without peripheral anterior synechia; (5) a slight decrease in vision, no visual field defect and normal optic disc at the early stage. Exclusion criteria included a history of ocular trauma, a history of ophthalmic herpes simplex virus (HSV) or cytomegalovirus (CMV), ocular laser treatment, or ophthalmic surgery before the diagnosis of PSS [10].

One hundred and twenty-eight unrelated healthy volunteer blood donors from Shenzhen Blood Center who had normal IOP and optic disc were recruited as control subjects. All control subjects had no KPs, no any history of eye diseases, and no any history of ophthalmic HSV or CMV. The age, sex and ethnic background of the control group were matched with the case group. All cases and controls were southern Han Chinese.

### DNA extraction

Peripheral blood samples (5 mL) were collected from each participant, and were anticoagulated with 5% EDTA and stored at -80°C. Genomic DNA was extracted from the peripheral blood with a TECAN DNA workstation (Sweden). DNA purity and concentration were tested using an Eppendorf spectral photometer.

### Sequencing-based typing for HLA class I and II

Genomic DNA was used for SBT with the commercial AlleleSEQR HLA SBT kit (Atria Genetics, San Francisco, CA) according the manufacturer’s instructions. PCR product was purified using ExoSAP-IT (Atria Genetics). Exons 2, 3 and 4 for HLA-A,-B,-C loci, exon 2 for HLA-DRB1 and exons 2 and 3 for HLA-DQB1 were sequenced in both directions on an ABI 3730 DNA Sequencer (Applied Biosystem, Foster City, CA). HLA genotypes were assigned in four-digit level with the help of the ASSIGN 3.5 software (Conexio Genomics, Applecross, Australia; S1 and S2 Figs). Synonymous mutations were not taken into account.

### Statistical analysis

Allele frequencies were calculated by direct counting. The difference in allele frequency and sex between cases and controls was tested by the χ^2^ test or Fisher’s exact test using SPSS (version 19.0, SPSS Inc., Chicago, IL). Independent-samples T test was used for the comparison of age and IOP between the two groups. Haplotype inference was performed using Arlequin V3.5 with the following settings: ELB algorithm performed at the haplotype level, burnin 100000, sampling interval 500, ave. prior alpha 0.01, and het. site influence zone 5. Multiple testings were corrected using the Bonferroni method and the corrected *P* value (*P*_*c*_) was calculated by multiplying the *P* value with the number of tests performed. *P*_*c*_ < 0.05 was considered statistically significant. Odds ratio (OR) and 95% confidence interval (CI) were estimated whenever applicable.

## Results

### Demographic and clinical features of patients and control subjects

The mean (SD) age of PSS patients (n = 100) was 32.6 (7.9) years ranging from 18 to 50 years. 47.0% of the patients were female and 53.0% were male. In the control group (n = 128), the mean (SD) age was 31.9 (7.3) years ranging from 19 to 50 years. 40.6% of the controls were female and 59.3% were male. No significant difference in age and sex was found between the patient group and control group (*P* = 0.58 and 0.34 respectively; Table 1). The mean (SD) IOP of eyes with PSS was 42.2 (7.1) mmHg while 15.3 (2.4) mmHg in the control group. The IOP of eyes with PSS was significantly higher than that in control subjects (*P* < 0.001; Table 1).

**Table 1:** The demographic and clinical features of the PSS cases and controls.

Feature	PSS (n = 100)	Controls (n = 128)	*P*
Age, mean (SD), year	32.6(7.9)	31.9(7.3)	0.58^a^
Sex (M/F)	53/47	76/52	0.34^b^
IOP, mean (SD), mmHg	42.2(7.1)	15.3(2.4)	<0.001^a^
KPs (Y/N)	Y	N	

Abbreviations: PSS, Posner-Schlossman syndrome; IOP, intraocular pressure;KPs, keratic precipitates.

^a^ Independent-samples T test

^b^ χ^2^ test.

### Allele frequencies of HLA-A,-B,-C,-DRB1 and-DQB1

The number of identified HLA-A,-B,-C,-DRB1 and-DQB1alleles were 20, 34, 20, 17 and 16 respectively in PSS patients. 16 HLA-A alleles, 38 HLA-B alleles, 22 HLA-C alleles, 16 HLA-DRB1 alleles and 16 HLA-DQB1 alleles were detected in control subjects (Tables 2 and 3). The genotype distributions of HLA-A,-B,-C-DRB1 and-DQB1 loci in both the patient and control groups were in accordance with Hardy–Weinberg equilibrium (*P* > 0.24; data not shown).

**Table 2:** Frequencies of HLA-A and-B alleles in PSS cases and controls.

HLA-A*	PSS	Controls	*P*	HLA-B*	PSS	Controls	*P*	HLA-B*	PSS	Controls	*P*
*0101*	2 (1.0)	2 (0.8)	0.80	*0705*	2 (1.0)	1 (0.4)	0.42	*3701*	0	2 (0.8)	0.34
*0201*	16 (8.0)	18 (7.0)	0.70	*0801*	1 (0.5)	1 (0.4)	0.86	*3801*	1 (0.5)	1 (0.4)	0.86
*0203*	5 (2.5)	16 (6.3)	0.06	*1301*	4 (2.0)	23 (9.0)	0.003	*3802*	5 (2.5)	8 (3.1)	0.69
*0206*	4 (2.0)	6 (2.3)	0.80	*1302*	1 (0.5)	4 (1.6)	0.28	*3901*	7 (3.5)	3(1.17)	0.09
*0207*	32 (16.0)	34 (13.3)	0.41	*1501*	4 (2.0)	12 (4.7)	0.12	*4001*	26 (13.0)	46 (18.0)	0.15
*0301*	0	4 (1.6)	0.12	*1502*	19 (9.5)	20 (7.8)	0.52	*4002*	4 (2.0)	1 (0.4)	0.10
*1101*	69 (34.5)	80 (31.3)	0.46	*1507*	0	1 (0.4)	0.59	*4003*	0	1 (0.4)	0.59
*1102*	8 (4.0)	12 (4.7)	0.72	*1511*	0	2 (0.8)	0.34	*4006*	1 (0.5)	7 (2.7)	0.07
*1136*	0	1 (0.4)	0.58	*1512*	2 (1.0)	0	0.17	*4403*	5 (2.5)	2 (0.8)	0.27
*2402*	26 (13.0)	38 (14.8)	0.57	*1518*	2 (1.0)	0	0.17	*4601*	41 (20.5)	39 (15.2)	0.14
*2403*	1 (0.5)	1 (0.4)	0.86	*1519*	0	1 (0.4)	0.59	*4801*	1 (0.5)	3 (1.2)	0.45
*2404*	1 (0.5)	0	0.37	*1521*	0	1 (0.4)	0.59	*5101*	13 (6.5)	7 (2.7)	0.05
*2407*	2 (1.0)	0	0.17	*1525*	2 (1.0)	0	0.17	*5102*	6 (3.0)	5 (2.0)	0.47
*2408*	1 (0.5)	0	0.37	*1527*	2 (1.0)	0	0.17	*5201*	0	3 (1.2)	0.20
*2410*	1 (0.5)	0	0.37	*1558*	0	1 (0.4)	0.59	*5401*	8 (4.0)	6 (2.3)	0.31
*2601*	3 (1.5)	6 (2.3)	0.52	*1801*	0	2 (0.8)	0.34	*5502*	11 (5.5)	8 (3.1)	0.21
*2901*	1 (0.5)	0	0.37	*1802*	1 (0.5)	0	0.37	*5601*	1 (0.5)	2 (0.8)	0.71
*3001*	2 (1.0)	4 (1.6)	0.61	*2704*	6 (3.0)	6 (2.3)	0.66	*5603*	1 (0.5)	0	0.37
*3101*	4 (2.0)	7 (2.7)	0.90	*2705*	2 (1.0)	1 (0.4)	0.42	*5604*	1 (0.5)	0	0.37
*3201*	1 (0.5)	3 (1.2)	0.45	*2706*	0	1 (0.4)	0.59	*5701*	1 (0.5)	0	0.37
*3303*	20 (10.0)	24 (9.4)	0.82	*2707*	0	1 (0.4)	0.59	*5801*	13 (6.5)	22 (8.6)	0.40
*6801*	1(0.5)	0	0.37	*3501*	2 (1.0)	4 (1.6)	0.60	*6701*	0	1 (0.4)	0.59
				*3503*	0	5 (2.0)	0.08	*7801*	1 (0.5)	0	0.37
				*3505*	3 (1.5)	2 (0.8)	0.46				

The allele frequencies were presented as allele count (%). Abbreviations: PSS, Posner-Schlossman syndrome; χ^2^ test was used.

**Table 3:** Frequencies of HLA-C,-DQB1 and-DQB1 alleles in PSS cases and controls.

HLA-C*	PSS	Controls	*P*	HLA-DRB1*	PSS	Controls	*P*	HLA-DQB1*	PSS	Controls	*P*
*0102*	55 (27.5)	51 (19.9)	0.06	*0101*	1 (0.5)	0	0.37	*0201*	10 (5.0)	14 (5.5)	0.82
*0103*	1 (0.5)	0	0.37	*0301*	10 (5.0)	14 (5.5)	0.82	*0202*	4 (2.0)	7 (2.7)	0.84
*0108*	0	1 (0.4)	0.59	*0401*	1 (0.5)	1 (0.4)	0.86	*0301*	46 (23.0)	52 (20.3)	0.49
*0202*	2 (1.0)	1 (0.4)	0.42	*0403*	2 (1.0)	6 (2.3)	0.28	*0302*	14 (7.0)	15 (5.9)	0.62
*0302*	13 (6.5)	21 (8.2)	0.49	*0404*	2 (1.0)	0	0.17	*0303*	44 (22.0)	46 (18.0)	0.28
*0303*	11 (5.5)	13 (5.1)	0.84	*0405*	13 (6.5)	21 (8.2)	0.49	*0401*	11 (5.5)	20 (7.8)	0.33
*0304*	19 (9.5)	39 (15.2)	0.07	*0406*	8 (4.0)	7 (2.7)	0.45	*0402*	1 (0.5)	3 (1.2)	0.45
*0401*	9 (4.5)	15 (5.9)	0.52	*0407*	0	1 (0.4)	0.59	*0501*	5 (2.5)	9 (3.5)	0.73
*0403*	1 (0.5)	3 (1.2)	0.45	*0701*	5 (2.5)	8 (3.1)	0.91	*0502*	19 (9.5)	28 (10.9)	0.62
*0406*	0	1 (0.4)	0.59	*0801*	1 (0.5)	0	0.37	*0503*	9 (4.5)	11 (4.3)	0.92
*0415*	0	1 (0.4)	0.59	*0802*	1 (0.5)	2 (0.8)	0.71	*0601*	20 (10.0)	30 (11.7)	0.56
*0602*	2 (1.0)	6 (2.3)	0.28	*0803*	14 (7.0)	14 (5.5)	0.50	*0602*	10 (5.0)	10 (3.9)	0.57
*0701*	1 (0.5)	1 (0.4)	0.86	*0809*	0	1 (0.4)	0.59	*0603*	1 (0.5)	2 (0.8)	0.71
*0702*	26 (13.0)	43 (16.8)	0.26	*0901*	42 (21.0)	45 (17.6)	0.36	*0604*	1 (0.5)	2 (0.8)	0.71
*0704*	2 (1.0)	1 (0.4)	0.86	*1001*	1 (0.5)	3 (1.2)	0.80	*0609*	4 (2.0)	5 (2.0)	0.97
*0801*	23 (11.5)	27 (10.6)	0.75	*1101*	7 (3.5)	10 (3.9)	0.82	*0610*	1 (0.5)	2 (0.8)	0.71
*0803*	1 (0.5)	1 (0.4)	0.86	*1104*	1 (0.5)	0	0.37				
*1202*	6 (3.0)	8 (3.1)	0.94	*1201*	10 (5.0)	8 (3.3)	0.31				
*1203*	3 (1.5)	8 (3.1)	0.26	*1202*	26 (13.0)	27 (10.6)	0.42				
*1402*	18 (9.0)	6 (2.3)	0.002	*1301*	0	2 (0.8)	0.34				
*1403*	2 (1.0)	1 (0.4)	0.42								
*1502*	3 (1.5)	7 (2.7)	0.37								
*1505*	2 (1.0)	0	0.17								
*1701*	0	1 (0.4)	0.59								

The allele frequencies were presented as allele count (%). Abbreviations: PSS, Posner-Schlossman syndrome; χ^2^ test was used.

The allele frequency of HLA-C*1402 in patients with PSS was significantly higher than that in control subjects (9.0% vs. 2.3%, *P* = 0.002, *P*_*c*_ = 0.04, OR = 4.12; Table 4). The allele frequency of HLA-B*1301 in the patient group was significantly lower than that in the control group (2.0% vs. 9.0%, *P* = 0.003, OR = 0.21; Table 4), although this association did not survive the Bonferroni correction (*P*_*c*_ = 0.16). No significant difference was found between PSS patients and controls for the frequencies of HLA-A,-DRB1 and-DQB1 alleles (*P* > 0.05).

**Table 4:** Significant alleles and haplotypes associated with PSS.

	PSS (2n = 200)		Controls (2n = 256)				
HLA-	Count	Frequency (%)	Count	Frequency (%)	*P*	*P*_*c*_	OR (95% CI)
Allele							
B*1301	4	2.0	23	9.0	0.003	0.16	0.21 (0.07–0.61)
C*1402	18	9.0	6	2.3	0.002	0.04	4.12 (1.60–10.59)
Haplotype							
A*1101~C*1402	10	5.0	3	1.2	0.03	0.84	4.44 (1.21–16.35)
B*5101~C*1402	12	6.0	5	2.0	0.02	0.52	3.20 (1.11–9.25)
B*1301~C*0304	4	2.0	21	8.2	0.007	0.16	0.23 (0.08–0.68)

Abbreviations: PSS, Posner-Schlossman syndrome; *P*_*c*_, Bonferroni corrected *P* value by multiplying the *P* value with the number of tests performed; CI, confidence interval; OR, odds ratio; χ^2^ test was used.

### Haplotypefrequencies of HLA-A,-B,-C,-DRB1 and-DQB1

The haplotype frequencies of HLA-A*1101~C*1402 and B*5101~C*1402 in PSS patients were significantly higher than that in controls (5.0% vs. 1.2%, *P* = 0.03, OR = 4.44; 6.0% vs. 2.0%, *P* = 0.02, OR = 3.20; respectively; Table 4), although these associations did not survive the Bonferroni correction (*P*_*c*_ > 0.52). The haplotype frequency of HLA-B*1301~C*0304 in PSS patients was significantly lower than that in controls (2.0% vs. 8.2%, *P* = 0.007, OR = 0.23; Table 4), although this association did not survive the Bonferroni correction (*P*_*c*_ = 0.16). No significant difference in the haplotype frequencies of the other loci was found between patients with PSS and control subjects (*P* > 0.05).

## Discussion

Conventional serological typing, PCR-based genotyping and direct sequencing of HLA genes have revealed more specific HLA alleles associated with various eye diseases. However, the association between PSS and HLA has been poorly investigated. To date, only one study by Hirose et al.^27^ reported that HLA-Bw54 and haplotype HLA-Bw54-Cw1 showed significant association with PSS in a small sample of the Japanese population. Despite of the limited sample size, the Japanese study suggests that immunogenetic factors closely associated with the major histocompatibility complex may play an important role in the pathogenesis of PSS. However, only class I HLA (HLA-A, B and C) were typed at low resolution level by serological method in the Japanese study [27]. In the present study, we for the first time investigated the association of class I and class II HLA diversity with PSS at high resolution level by sequencing based typing in a larger sample of southern Chinese Han population.

In the present study, we found that the allele frequency of HLA-C*1402 in patients with PSS was significantly higher than that in the controls (OR = 4.12, 95% CI: 1.60–10.59; Table 4), suggesting that HLA-C*1402 might be a risk factor for PSS. However, the allele frequency of HLA-B*1301 in PSS patients was significantly lower than that in the control group (OR = 0.21, 95% CI: 0.07–0.61; Table 4), suggesting that HLA-B*1301 might be a protective factor for PSS. We did not find significant difference in allele frequencies of the HLA-A,-DRB1 and-DQB1 loci between the PSS group and the control group (Tables 2 and 3), indicating that these HLA loci might not contribute to the development of PSS.

HLA-B*1301~C*0304 is a common haplotype in our samples of southern Chinese Han population, with a relatively high frequency of 8.2% (Table 4). Notably, the HLA-B*1301 allele was in strong linkage disequilibrium with the HLA-C*0304 allele. Among a total of 23 HLA-B*1301 alleles identified in the control group, 21 alleles linked with HLA-C*0304, and all the 4 HLA-B*1301 alleles in the PSS group linked with HLA-C*0304. Our results showed that the haplotype frequency of HLA-B*1301~C*0304 in PSS patients was significantly lower than that in controls PSS (OR = 0.23; 95% CI: 0.08–0.68; Table 4), indicating that individuals carrying this haplotype might have a reduced risk to develop PSS.

In this study, we identified two high-risk haplotypes, HLA-A*1101~C*1402 and B*5101~C*1402 (OR = 4.44 and 3.20 respectively; Table 4). Intriguingly, both of the haplotypes were associated with HLA-C*1402, which is in accordance with that HLA-C*1402 was associated with increased risk of developing PSS.

The HLA-B27 allele was relatively rare in our study, accounting for only 4.0% (3.0% HLA-B*2704 and 1.0% HLA-B*2705) of the PSS patients and 3.5% (2.3% HLA-B*2704, 0.4% HLA-B*2705, 0.4% HLA-B*2706 and 0.4% HLA-B*2707) of the controls (Table 2). In contrast to the findings that HLA-B27 is associated with acute anterior uveitis [14–17], our results showed that HLA-B27 was not significantly associated with PSS, suggesting that PSS and AAU might be different uveitis. In addition, we did not find significant associations between PSS and HLA-DRB1or HLA-DQ B1, which have been associated with POAG in Mexicans [25]. Collectively, these findings indicate that there might be different pathogenesis between PSS and AAU or POAG.

It has been reported that ophthalmic HSV or CMV infection might be associated with PSS [5–7, 29]. In the present study, all cases and control subjects had no any history of ophthalmic HSV or CMV. Therefore, our findings that the HLA-B and HLA-C polymorphisms are associated with PSS are unlikely to be confounded by the possible association of ophthalmic HSV or CMV infection with PSS.

## Conclusions

In summary, the present study for the first time demonstrated that the allelic polymorphisms of HLA were nominally associated with PSS in southern Chinese Han population. Our results suggest that gene polymorphisms at the HLA-B and HLA-C loci might contribute to developing PSS but even larger datasets are required to confirm these findings. Specifically, HLA-C*1402, A*1101~C*1402 and B*5101~C*1402 might predispose to PSS, while HLA-B*1301, especially B*1301~C*0304 might be protective against PSS. These findings will provide valuable new clues for investigation into the mechanisms and development of new diagnosis and treatment for PSS.

## Supporting Information

S1 Fig One assignment of class I HLA genotypes by sequencing based typing. (TIF)

S2 Fig One assignment of class II HLA genotypes by sequencing based typing. (TIF)

## References

[1] Posner A, Schlossman A. Syndrome of unilateral recurrent attacks of glaucoma with cyclitic symptoms. Arch Ophthalmol. 1948;39: 517–535. 10.1001/archopht.1948.00900020525007 18123283

[2] Jap A, Sivakumar M, Chee SP. Is Posner Schlossman syndrome benign? Ophthalmology. 2001;108: 913–918. 11320022 10.1016/s0161-6420(01)00551-6

[3] Kim TH, Kim JL, Kee C.Optic disc atrophy in patient with Posner-Schlossman syndrome. Korean J Ophthalmol. 2012;26: 473–477. 10.3341/kjo.2012.26.6.473 23204806 PMC3506825

[4] Knox DL.Glaucomatocyclitic crises and systemic disease: peptic ulcer, other gastrointestinal disorders, allergy and stress. Trans Am Ophthalmol Soc. 1988;86: 473–495. 2979027 PMC1298821

[5] Takusagawa HL, Liu Y, Wiggs JL. Infectious theories of Posner-Schlossman syndrome. Int Ophthalmol Clin. 2011;51: 105–115. 10.1097/IIO.0b013e31822d6ab4 21897144

[6] Chee SP, Jap A. Presumed Fuchs heterochromic iridocyclitis and Posner-Schlossman syndrome: comparison of cytomegaloviruspositive and negative eyes. Am J Ophthalmol. 2008;146: 883–889. 10.1016/j.ajo.2008.09.001 19027423

[7] Teoh SB, Thean L, Koay E. Cytomegalovirus in aetiology of Posner-Schlossman syndrome: evidence from quantitative polymerase chain reaction. Eye. 2005;19: 1338–1340. 15543169 10.1038/sj.eye.6701757

[8] van Boxtel LA, van der Lelij A, van der Meer J, Los LI. Cytomegalovirus as a cause of anterior uveitis in immunocompetent patients. Ophthalmology. 2007;114: 1358–1362. 17296229 10.1016/j.ophtha.2006.09.035

[9] Sacca SC, Pascotto A, Camicione P, Capris P, Izzotti A. Oxidative DNA damage in the human trabecular meshwork: clinical correlation in patients with primary open-angle glaucoma. Arch Ophthalmol. 2005;123: 458–463. 15824217 10.1001/archopht.123.4.458

[10] Shen SC, Ho WJ, Wu SC, Lin HC, Lin YS, Tsay PK, et al Peripheral vascular endothelial dysfunction in glaucomatocyclitic crisis: a preliminary study. Invest Ophthalmol Vis Sci. 2010;51: 272–276. 10.1167/iovs.09-3849 19684007

[11] Su WW, Cheng ST, Hsu TS, Ho WJ. Abnormal flow-mediated vasodilation in normal tension glaucoma using a noninvasive determination for peripheral endothelial dysfunction. Invest Ophthalmol Vis Sci. 2006;47: 3390–3394. 16877407 10.1167/iovs.06-0024

[12] Su WW, Cheng ST, Ho WJ, Tsay PK, Wu SC, Chang SH. Glaucoma is associated with peripheral vascular endothelial dysfunction. Ophthalmology. 2008;115: 1173–1178. 18076992 10.1016/j.ophtha.2007.10.026

[13] Flammer J, Orgül S, Costa VP, Orzalesi N, Krieglstein GK, Serra LM, et al The impact of ocular blood flow in glaucoma. Prog Retin Eye Res. 2002;21: 359–393. 12150988 10.1016/s1350-9462(02)00008-3

[14] Yabuki K, Inoko H, Ohno S. HLA testing in patients with uveitis. Int Ophthalmol Clin. 2000;40: 19–35. 10791255 10.1097/00004397-200004000-00004

[15] Chung YM, Liao HT, Lin KC, Lin YC, Chou CT, Chen CH, et al Prevalence of spondyloarthritis in 504 Chinese patients with HLA-B27-associated acute anterior uveitis. Scand J Rheumatol. 2009;38: 84–90. 10.1080/03009740802385423 18821178

[16] Mielants H, Veys EM, Verbraeken H, De Vos M, Cuvelier C. HLAB27 positive idiopathic acute anterior uveitis: a unique manifestation of subclinical gut inflammation. J Rheumatol. 1990;17: 841–842. 2388209

[17] Chang JH, McCluskey PJ, Wakefield D. Acute anterior uveitis and HLA-B27. Surv Ophthalmol. 2005;50: 364–388. 15967191 10.1016/j.survophthal.2005.04.003

[18] Levinson RD, Rajalingam R, Park MS, Reed EF, Gjertson DW, Kappel PJ, et al Human leukocyte antigen A29 subtypes associated with birdshot retinochoroidopathy.Am J Ophthalmol. 2004;138: 631–634. 15488792 10.1016/j.ajo.2004.06.016

[19] Zamecki KJ1, Jabs DA. HLA typing in uveitis: use and misuse. Am J Ophthalmol. 2010;149: 189–193. 10.1016/j.ajo.2009.09.018 20103052

[20] Donvito B1, Monnet D, Tabary T, Delair E, Vittier M, Réveil B, et al A new HLA extended haplotype containing the A*2910 allele in birdshot retinochoroidopathy: susceptibility narrowed to the HLA molecule itself. Invest Ophthalmol Vis Sci. 2010;51: 2525–2528. 10.1167/iovs.09-4329 19959637

[21] LeHoang P, Ozdemir N, Benhamou A, Tabary T, Edelson C, Betuel H, et al HLA-A29.2 subtype associated with birdshot retinochoroidopathy. Am J Ophthalmol. 1992;113: 33–35. 1728143 10.1016/s0002-9394(14)75749-6

[22] Oruc S, Kaplan AD, Galen M, Kaplan HJ. Uveitis referral pattern in a Midwest University Eye Center. Ocul Immunol Inflamm. 2003;11: 287–298. 14704900 10.1076/ocii.11.4.287.18270

[23] Kang EH, Kim JY, Takeuchi F, Kim JW, Shin K, Lee EY, et al Associations between the HLA-A polymorphism and the clinical manifestations of Behcet's disease. Arthritis Res Ther. 2011;13: R49 Available: http://arthritis-research.com/content/13/2/R49. 10.1186/ar3292 21429233 PMC3132038

[24] Gil-Carrasco F, Granados J, Barojas-Weber E, Gilbert-Lucido ME, Vargas-Alarcón G. Immunogenetic aspects in primary open-angle glaucoma in family members of Mexican mestizo glaucomatous patients. Am J Ophthalmol. 1994;118: 744–748. 7977600 10.1016/s0002-9394(14)72553-x

[25] Gil-Carrasco F, Vargas-Alarcón G, Zúñiga J, Tinajero-Castañeda O, Hernández-Martinez B, Hernández-Pacheco G, et al HLA-DRB and HLA-DQB loci in the genetic susceptibility to develop glaucoma in Mexicans. Am J Ophthalmol. 1999;128: 297–300. 10511023 10.1016/s0002-9394(99)00180-4

[26] Ferreri G, D'Andrea A, Castagna I, Rechichi C, Pettinato G, D'Andrea D. The role of class I and class II HLA antigens in primary open angle glaucoma (POAG). Acta Ophthalmol Scand Suppl. 1998;(227): 17–19. 9972328 10.1111/j.1600-0420.1998.tb00866.x

[27] Hirose S, Ohno S, Matsuda H. HLA-Bw54 and glaucomatocyclitic crisis. Arch Ophthalmol. 1985;103: 1837–1839. 4074175 10.1001/archopht.1985.01050120071023

[28] Shazly TA, Aljajeh M, Latina MA. Posner-Schlossman glaucomatocyclitic crisis. Semin Ophthalmol. 2011;26: 282–284. 10.3109/08820538.2011.605821 21958175

[29] Yamamoto S, Pavan-Langston D, Tada R, Yamamoto R, Kinoshita S, Nishida K, et al Possible role of herpes simplex virus in the origin of Posner-Schlossman syndrome. Am J Ophthalmol. 1995;119: 796–798. 7785697 10.1016/s0002-9394(14)72788-6

