# Supplementary figures and images for: Human Leukocyte Antigens-B and -C Loci Associated with Posner-Schlossman Syndrome in a Southern Chinese Population
Source: PLoS One. 2015 Jul 10;10(7):e0132179. doi: 10.1371/journal.pone.0132179 (PMC4498812; doi:10.1371/journal.pone.0132179)

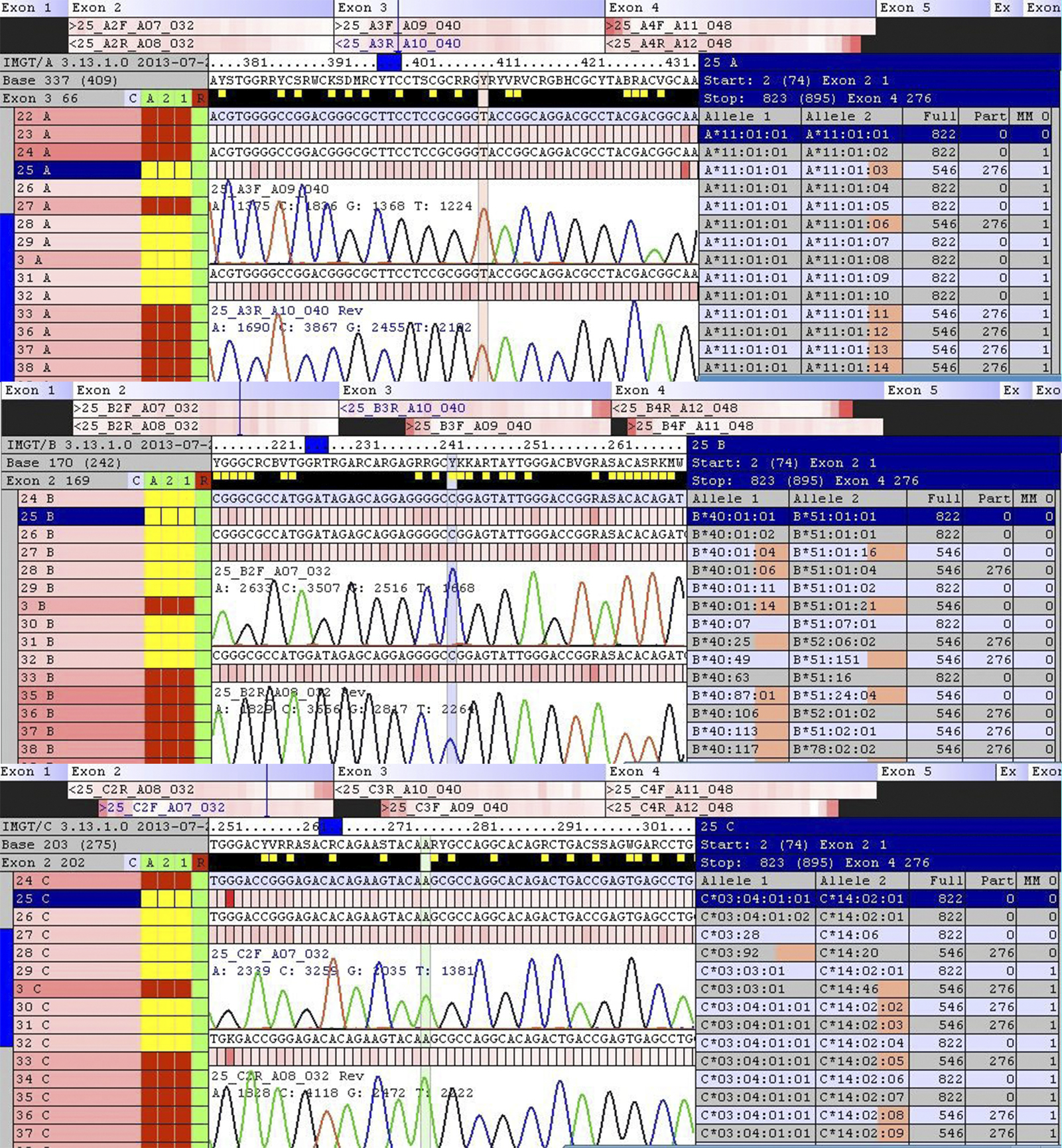

Supplement: S1 Fig — (TIF) [file pone.0132179.s001.tif]

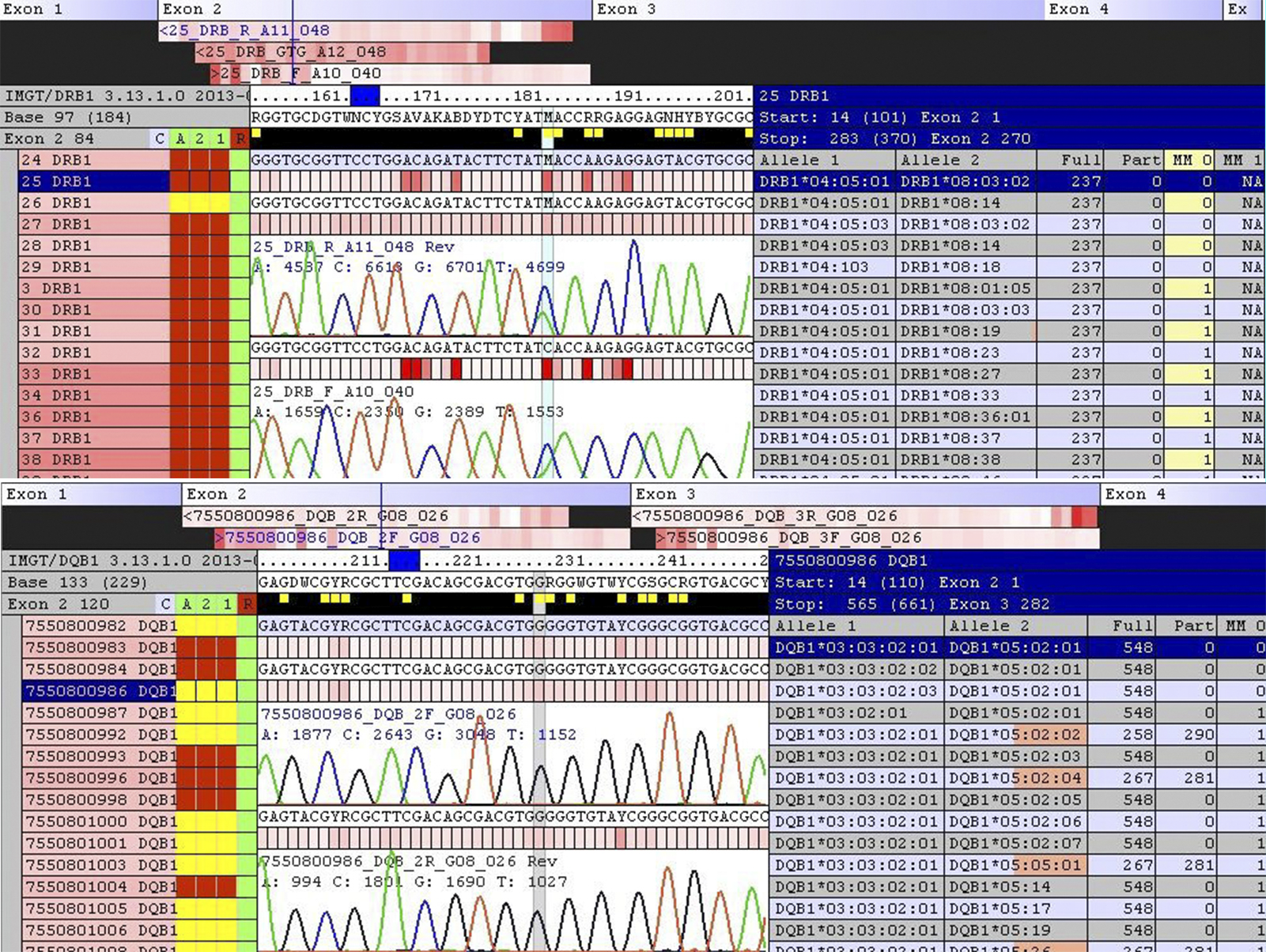

Supplement: S2 Fig — (TIF) [file pone.0132179.s002.tif]
